# Fire and termite resistance of wood treated with PF_6_-based ionic liquids

**DOI:** 10.1038/s41598-022-18792-7

**Published:** 2022-08-25

**Authors:** Hisashi Miyafuji, Koji Minamoto

**Affiliations:** Graduate School of Life and Environmental Sciences, Kyoto Prefectural University, 1-5 Hangi-cho, Shimogamo, Sakyo-ku, Kyoto, 606-8522 Japan

**Keywords:** Composites, Ionic liquids

## Abstract

Six PF_6_-based ionic liquids (ILs) were investigated to evaluate their potential as chemicals for enhancing fire and termite resistance of wood. The ILs used in this study included 1-methyl-1-propylpyrrolidinium hexafluorophosphate ([MPPL]PF_6_), 1-methyl-1-propylpiperidinium hexafluorophosphate ([MPPR]PF_6_), 1-ethyl-3-methylimidazolium hexafluorophosphate ([EMIM]PF_6_), tetrabutylphosphonium hexafluorophosphate ([TBP]PF_6_), trihexyltetradecylphosphonium hexafluorophosphate ([THP]PF_6_), and 1-butylpyridinium hexafluorophosphate ([BPYR]PF_6_). All of the IL-treated wood samples did not undergo any morphological changes, and exhibited enhanced fire- and termite resistance compared with untreated wood. The fire resistance properties of all of the prepared IL-treated wood specimens were comparable. However, the [EMIM]PF_6_- and [THP]PF_6_-treated wood showed slightly inferior termite resistance among the tested IL-treated woods. Overall, [TBP]PF_6_ was the most promising candidate among the evaluated PF_6_-based ILs because it is stable in wood without leaching after water penetration.

## Introduction

Wood is widely used as a building material. However, the properties of wood, e.g., its combustibility, dimensional stability, and biodeterioration must be managed for practical use. To mitigate these disadvantageous characteristics, numerous wood preservatives have been developed.

Boric acid, phosphoric acid, and their salts are known to enhance the fire resistance of wood or wood-based materials^1–13^. Treatments with colloidal silicate^14,15^ or metal oxides via sol–gel processes^16–25^ have also been reported to endow wood-inorganic composites with fire-resistant properties.

It has been demonstrated that acetylation and formalization are effective techniques for improving the dimensional stability of wood^26–28^. The hydrophilic hydroxyl groups in the wood (which acts as an acceptor of water molecules from external moisture) are replaced with hydrophobic acetyl groups following the acetylation treatment. The elimination of hydroxyl groups renders the acetylated wood hydrophobic and prevents water from being contained within the wood cells, thereby improving its dimensional stability. The dimensional changes in formalized wood are reduced by cross-linking the hydroxyl groups in the wood cell walls with formaldehyde.

Chromized arsenical copper (CCA) has primarily been used as a preservative to improve the biodeterioration of wood. However, CCA contains carcinogenic hexavalent chromium and arsenic, and large amounts of construction waste after using CCA-treated wood can lead to serious environmental pollution^29^; as a result, the production of CCA-treated wood has dropped sharply in recent years. In addition to CCA, organophosphorus and boric acid-derived compounds are known to enhance the biodeterioration of wood.

Recent research efforts have focused on ionic liquids (ILs) as alternative materials to improve the performance of wood. ILs are salts with melting points close to ambient temperature^30^. It was reported that treatment with didecyldimethylammonium chloride (DDAC) or didecyldimethylammonium tetrafluoroborate (DBF) can be effective against termites^31^, mold, and fungi^32^. In addition, 1-methyl-3-octylalkoxyimidazolium tetrafluoroborate, 1-methyl-3-nonylalkoxyimidazolium tetrafluoroborate^33^, 1-decyloxymethyl-4-dimethylaminopyridinium chloride, and 1-decyloxymethyl-4-dimethylaminopyridinium acesulfamates^34^ have been shown to improve the biodeterioration of wood. In another study, it was reported that 1-ethyl-3-methylimidazolium hexafluorophosphate enhanced the fire resistance properties of wood^35^. However, to our knowledge, there are still a limited number of studies investigating ILs as chemicals for enhancing fire and termite resistance of wood.

Therefore, this study evaluated the potential of various PF_6_-based ionic liquids as chemicals for enhancing fire and termite resistance of wood by measuring the fire- and termite-resistant properties of wood samples treated with these PF_6_-based ionic liquids.

## Results and discussion

### IL-treated woods

**Figure 1:**
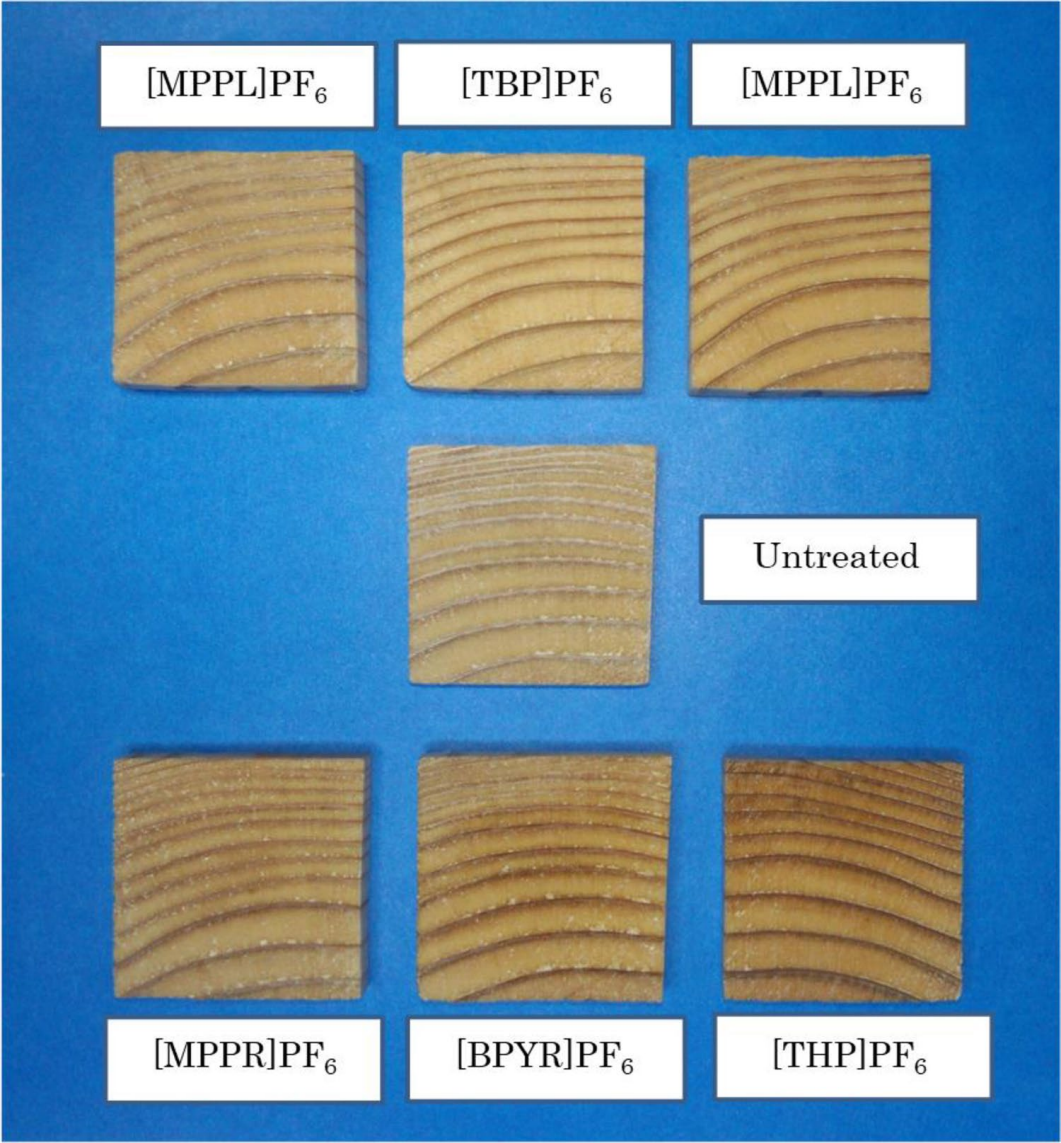
The appearance of IL-treated woods studied in this work.

Figure 1 shows the appearance of untreated and IL-treated wood samples. No warpage or cracks were observed in the various IL-treated woods, and no significant color change was detected. Table 2 presents the weight percent gain (WPG) and bulking coefficient (B) for the evaluated IL-treated woods. In all of the ionic liquids, the WPG values were positive, indicating that the ionic liquids were successfully impregnated into the wood. The WPG varied from 22 to 27%, which suggested that there did not seem to be much difference among the tested ILs in terms of WPG. These results revealed that among the ionic liquids used in this study, the structure of the cation had a negligible impact on the degree of impregnation into wood. The [MPPL]PF_6_, [MPPR]PF_6_, [EMIM]PF_6_, and [BPYR]PF_6_ ILs had positive values of B between 3.0 and 4.5%. This result indicated that these reagents were impregnated into the cell walls of the wood specimens. In contrast, [TBP]PF_6_ and [THP]PF_6_ had negative B values, indicating that the volume of the wood decreased after the IL-treatment. It is likely that [TBP]PF_6_ and [THP]PF_6_ were impregnated into the cell lumen, but did not penetrate into the cell wall; this would cause the cells to shrink when the ionic liquid aggregated in the cell lumen during the drying process. Thus, it was concluded that the structure of the cation in the PF_6_-based ionic liquids used in this study had a significant influence on B.

**Figure 2:**
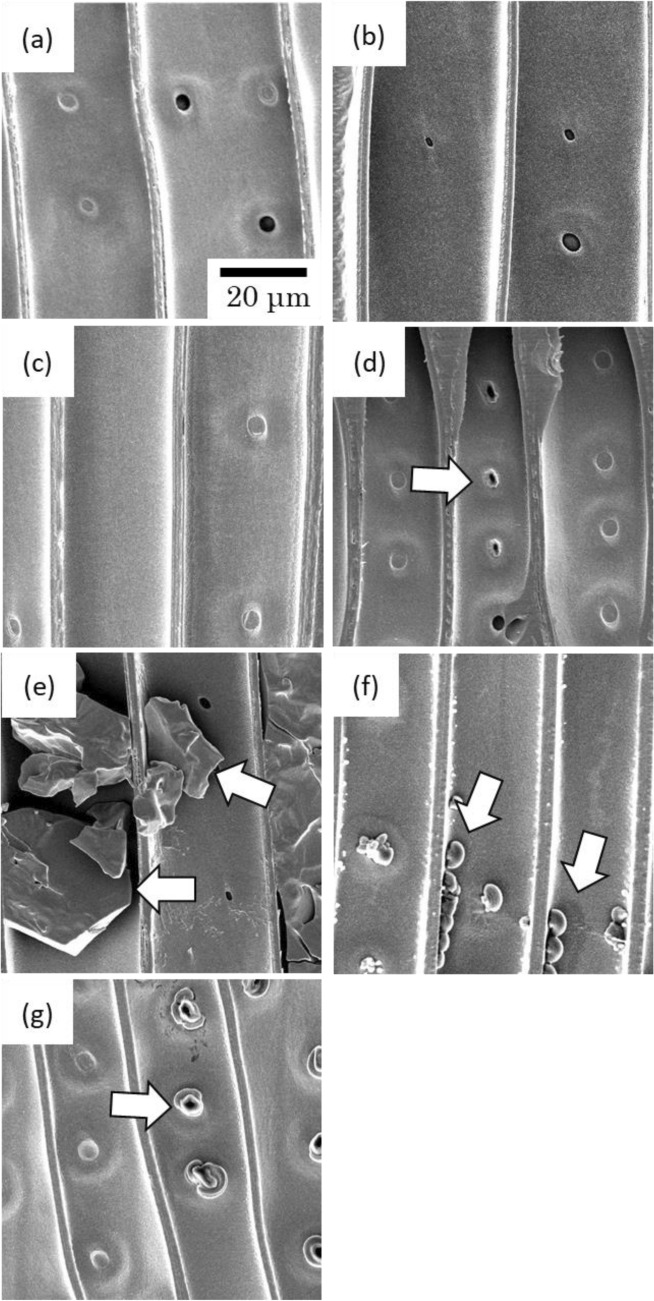
SEM micrographs of radial sections of IL-treated woods: (**a**) untreated; (**b**) [MPPL]PF_6_; (**c**) [MPPR]PF_6_; (**d**) [EMIM]PF_6_; (**e**) [TBP]PF_6_; (**f**) [THP]PF_6_; (**g**) [BPYR]PF_6_. arrows indicate ionic liquid.

Figure 2 shows the SEM micrographs of radial sections of the IL-treated wood specimens. No ionic liquids were detected in the [MPPL]PF_6_- and [MPPR]PF_6_-treated wood (Fig. 2b,c). In the [EMIM]PF_6_-treated wood, a very small amount of ionic liquid was observed in the pits, indicated by an arrow in Fig. 2d. In contrast, the micrographs of [TBP]PF_6_- and [THP]PF_6_-treated wood show significant amounts of ionic liquids in the cell lumen, as indicated by the arrows in Fig. 2e,f. In [TBP]PF_6_-treated wood, the ionic liquid existed as a block-shaped mass, whereas in [THP]PF_6_-treated wood, the IL was observed as lumps. In [BPYR]PF_6_-treated wood, a small round-shaped IL mass was observed around the pits. These results indicated that [MPPL]PF_6_, [MPPR]PF_6_, [EMIM]PF_6_, and [BPYR]PF_6_ were impregnated mainly into the cell wall. In contrast, [TBP]PF_6_ and [THP]PF_6_ were impregnated into the cell lumen. These results are consistent with the corresponding B values shown in Table 1.

**Table 1:** Ionic liquids used in this study.

Ionic Liquid	Abbreviation	Structure	Supplier
1-Methyl-1-propylpyrrolidinium hexafluorophosphate	[MPPL]PF_6_		Kanto chemical Co. Inc
1-Methyl-1-propylpiperidinium hexafluorophosphate	[MPPR]PF_6_		Kanto chemical Co. Inc
1-Ethyl-3-methylimidazolium hexafluorophosphate	[EMIM]PF_6_		Tokyo chemical industry Co,. Ltd
Tetrabutylphosphonium hexafluorophosphate	[TBP]PF_6_		Wako pure chemical industries, Ltd
Trihexyltetradecylphosphonium hexafluorophosphate	[THP]PF_6_		Tokyo chemical industry Co,. Ltd
1-Butylpyridinium hexafluorophosphate	[BPYR]PF_6_		Wako pure chemical industries, Ltd

### Antiswelling efficiency

Table 2 shows the ASE of various IL-treated wood samples. The [EMIM]PF_6_-treated wood had the highest value (38.0%) among the investigated IL-treated woods. The ASEs of [MPPL]PF_6_-, [MPPR]PF_6_-, and [BPYR]PF_6_-treated woods were in the range of 20–30%, whereas the ASEs of [TBP]PF_6_- and [THP]PF_6_-treated wood were significantly smaller (1.4% and 0.3%, respectively). The ASEs of these two types of IL-treated woods were low because a large amount of ionic liquid was present in the cell lumens, leading to the negative B values (Fig. 2 and Table 2). Because the IL-treated woods had a wide range of ASEs, it was concluded that the dimensional stability of these treated woods was not due to PF_6_^−^ (the common anion of all ionic liquids used in this study), but rather, it was determined by the IL cation.

**Table 2:** Weight percent gain, bulking coefficient, antiswelling efficiency, and leachability of IL-treated woods.

Ionic liquid	WPG(%)	B(%)	ASE(%)	Leachability(%)
[MPPL]PF_6_	24.4	3.3	28.4	73.8
[MPPR]PF_6_	23.3	3.0	24.0	59.8
[EMIM]PF_6_	25.2	4.5	38.0	73.5
[TBP]PF_6_	22.1	− 0.1	1.4	2.7
[THP]PF_6_	23.2	− 0.9	0.3	2.5
[BPYR]PF_6_	26.7	3.1	22.6	53.5

### Leachability of ILs

Table 2 also presents the leachability of the PF_6_-based ILs from the corresponding IL-treated woods. The leachabilities of [MPPL]PF_6_ and [EMIM]PF_6_ were 73.8% and 73.5%, respectively, which were the highest among the ionic liquids tested in this study. The [MPPR]PF_6_ and [BPYR]PF_6_ ILs also exhibited high leachabilities (59.8% and 53.5%, respectively). In these cases, since more than half of the impregnated ionic liquid was leached out, these four ionic liquids are considered liable to be leached from IL-treated woods. In contrast, [TBP]PF_6_ and [THP]PF_6_ showed much smaller leachability values (2.7% and 2.5%, respectively). Therefore, these ionic liquids are unlikely to leach from IL-treated woods. It is reasonable to conclude that these ionic liquids do not dissolve in water because they have long alkyl chains in their molecular structures, which give them hydrophobic properties. Overall, [TBP]PF_6_ and [THP]PF_6_ are expected to be effective chemicals for enhancing fire and termite resistance of wood without leaching from wood for a long time.

### Thermal properties of IL-treated woods

**Figure 3:**
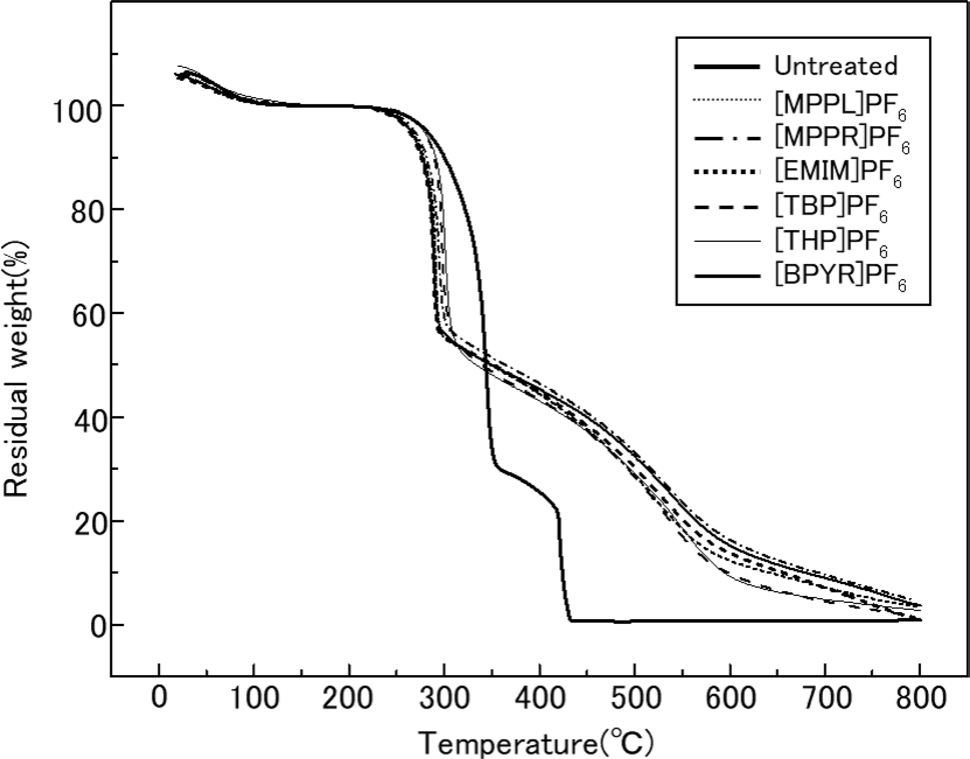
Thermogravimetric curves of the prepared IL-treated woods.

Figure 3 shows the thermogravimetric (TG) curves of the prepared IL-treated woods compared with that of untreated wood. For the untreated wood, approximately 75% of its weight loss occurred between 300 and 350 °C. Subsequently, further weight loss was observed from 400 to 450 °C, essentially reduced the residual weight to 0%. The TG curves of the IL-treated woods clearly differed from that of untreated wood. However, similar TG curves were obtained for all of the IL-treated wood samples tested in this study. The temperature range associated with abrupt weight loss was between 300 and 350 °C, which was shifted to a lower temperature relative to the first weight loss event in untreated wood. The residual weight following this reduction was approximately 50%. Subsequently, the weights of IL-treated samples decreased more gradually than that of untreated wood, and the residual weight reached ~ 40% at approximately 450 °C. At higher temperatures, the weight decreased gradually without any abrupt weight loss. The residual weights of the IL-treated woods at 800 °C reached 3.4%, 4.6%, 1.1% 1.8%, 2.7%, and 3.7% for [MPPL]PF_6_, [MPPR]PF_6_, [EMIM]PF_6_, [TBP]PF_6_, [THP]PF_6_, and [BPYR]PF_6_, respectively. Thus, it was concluded that all ILs suppressed weight loss due to burning of the wood.

**Figure 4:**
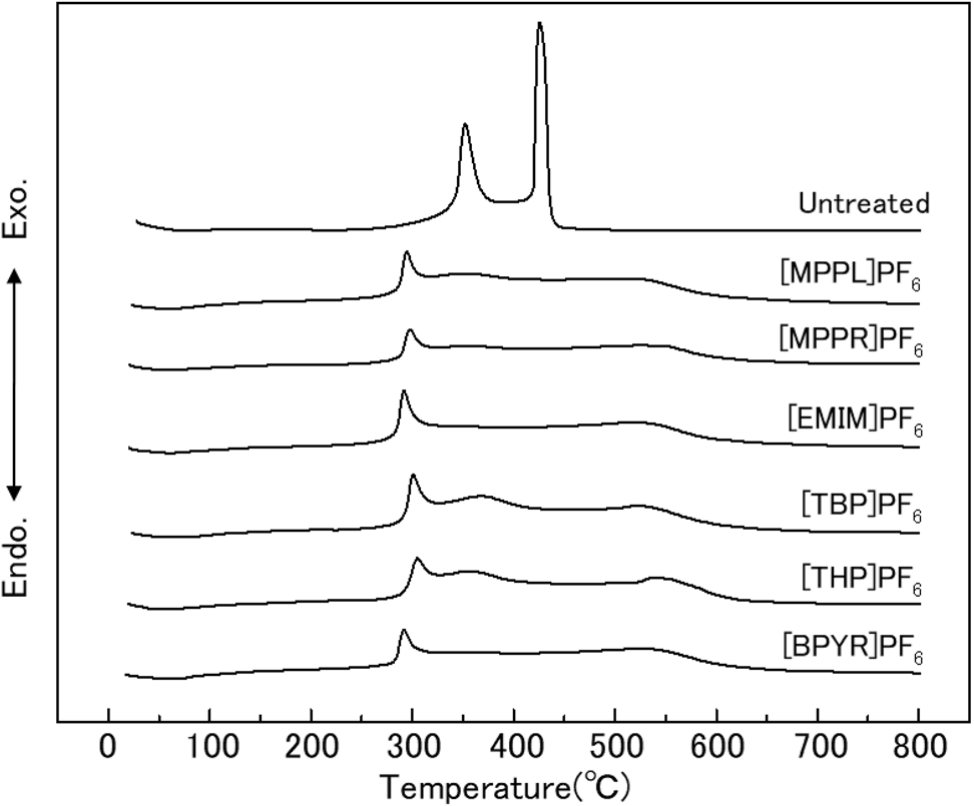
Differential thermal analysis curves of the prepared IL-treated woods.

Figure 4 shows the differential thermal analysis (DTA) curves of the prepared IL-treated woods, as well as untreated wood. For untreated wood, large peaks are observed at approximately 350 °C and 430 °C. These temperatures correspond to the two-step weight loss temperatures recorded on the TG curve for this sample (Fig. 3). The DTA curves of all IL-treated woods were generally the same. The peak observed at 350 °C in the untreated wood shifted to ~ 300 °C and decreased in intensity, indicating that heat generation was suppressed. Above 300 °C, a broad curve was observed, spanning temperatures up to 550 °C, but without any large or sharp features. It was therefore concluded that all IL treatments suppressed the burning of wood. From the results presented in Figs. 3 and 4, all of the ILs tested in this study enhanced the fire resistance of wood. Furthermore, since much differences were not seemed to be observed among the IL-treated woods in terms of thermal properties (as evidenced by their similar TG and DTA curves), it is reasonable to conclude that the fire resistance effect is not due to the cation, but rather, it is due to the anion PF_6_^−^.

### Termite resistance of IL-treated woods

**Figure 5:**
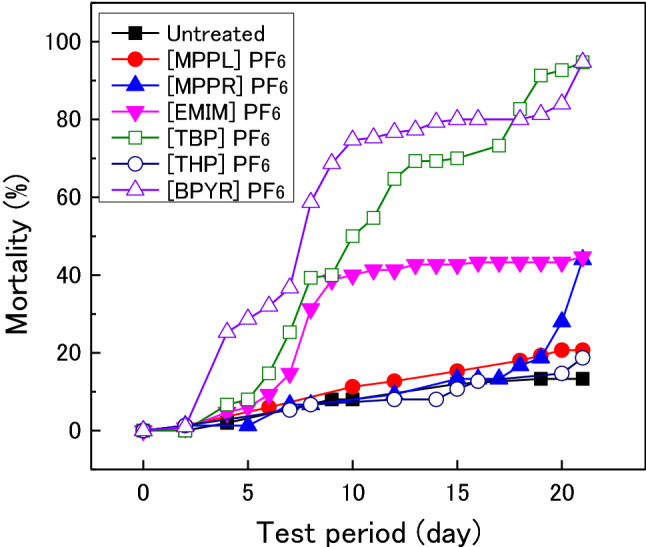
Changes in the mortality of *Coptotermes formosanus* (Shiraki) on IL-treated woods.

Figure 5 shows the changes in the mortality of *Coptotermes formosanus* for various IL-treated woods compared with that for untreated wood during the termite resistance test. For untreated wood, the mortality reached 13% after 21 days. For [TBP]PF_6_- and [BPYR]PF_6_-treated woods, the mortality began to increase after approximately five days and reached > 90% after 21 days. The mortality on [EMIM]PF_6_-treated wood also started to increase starting around day-five; however, the mortality only reached ~ 40% even after 21 days. For [MPPR]PF_6_-treated woods, the mortality began to increase sharply after 20–21 days. Much differences were not seemed to be observed among the other IL-treated woods, relative to the untreated wood.

**Figure 6:**
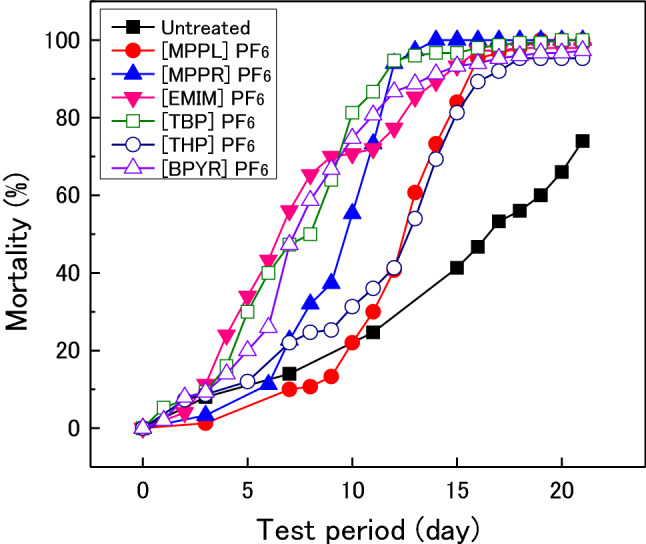
Changes in the mortality of *Reticulitermes speratus* (Kolbe) on IL-treated woods.

Figure 6 shows the changes in the mortality of *Reticulitermes speratus* for untreated and IL-treated woods during the termite resistance test. With untreated wood, the mortality reached 74% after 21 days. For [MPPR]PF_6_-, [EMIM]PF_6_-, [TBP]PF_6_-, and [BPYR]PF_6_-treated woods, the termite mortality began to increase soon after beginning the test, and exceeded 80% after 14 days. The mortality on [MPPL]PF_6_- and [THP]PF_6_-treated wood started to increase after 10 days. After 17 days, the mortality on all IL-treated woods exceeded 90%.

Table 3 shows the weight loss of various IL-treated woods after the termite resistance tests. The weight loss of the untreated wood after the *Coptotermes formosanus* resistance test was 12.2%, whereas the weight loss of IL-treated woods (except for [THP]PF_6_-treated wood) were essentially 0%. Even for [THP]PF_6_-treated wood, the weight loss only reached 2.1%, which was much lower than that of the untreated wood. The weight loss of untreated wood after the *Reticulitermes speratus* resistance test was 6.4%, whereas that of the IL-treated woods only reached up to 1.2%. The results in Figs. 5 and 6, and Table 3 confirm that the IL-treated woods exhibited termite resistance. The ionic liquids used in this study were proposed to suppress the feeding damage to wood caused by *Coptotermes formosanus* and *Reticulitermes speratus*; however, the effect of ILs on termite resistance differed depending on the structure of the cation. As shown in Fig. 5, the termite mortality on [TBP]PF_6_- and [BPYR]PF_6_-treated woods increased rapidly relative to that on untreated wood. The results in Table 3 and Fig. 5 indicate that [TBP]PF_6_ and [BPYR]PF_6_ are highly toxic to *Coptotermes formosanus*. The mortality rates on [MPPL]PF_6_- and [MPPR]PF_6_-treated wood were slightly higher than that on untreated wood in the latter half of the termite resistance test (Fig. 5). Additionally, [MPPL]PF_6_ and [MPPR]PF_6_ effectively prevented *Coptotermes formosanus* from eating, and because of this effect, the termites did not eat these IL-treated woods and starved as a result. The [THP]PF_6_ was also believed to have a similar effect; however, its effect was relatively lower because the termite mortality on [THP]PF_6_-treated wood was at the same level as on untreated wood (Fig. 5), and its weight loss after the termite resistance test was higher than that of [MPPL]PF_6_- and [MPPR]PF_6_-treated woods. [EMIM]PF_6_ is believed to both be toxic toward termites and prevent termites from eating because the mortality on [EMIM]PF_6_-treated wood increased rapidly in the first half of the termite resistance test and then continued to increase moderately, similar to the trend on untreated wood (Fig. 5). In the case of *Reticulitermes speratus*, although the differences in termite mortality trends among the studied IL-treated woods were not as pronounced (Fig. 6), similar effects as those described for *Coptotermes formosanus* are believed to be applicable. However, further accurate investigations on the resistance of IL-treated woods to *R speratus* are thought to be necessary. Consequently, among the ILs evaluated in this study, [TBP]PF_6_ and [BPYR]PF_6_ are the most effective in terms of enhancing the termite resistance of wood, and they can be considered as good termiticides.

**Table 3:** Weight loss of IL-treated woods after termite tests.

	Weight loss (%)	
Ionic liquid	*Coptotermes formosanus*	*Reticulitermes speratus*
Untreated	12.2	6.4
[MPPL]PF_6_	0.0	0.3
[MPPR]PF_6_	0.0	0.4
[EMIM]PF_6_	0.6	0.4
[TBP]PFe	0.1	0.4
[THP]PF_6_	2.1	1.2
[BPYR]PF_6_	0.1	0.3

## Materials and methods

### Chemicals

Ethanol, benzene and methanol were purchased from FUJIFILM Wako Pure Chemical Corporation (Osaka, Japan) and used without further purification. The ionic liquids used in this study are listed in Table 1; all ionic liquids were commercially available and used without further purification.

### Wood specimens

Specimens [30 mm (radial) × 30 mm (tangential) × 5 mm (longitudinal)] obtained from the sapwood portions of wild type of cultivated Japanese cedar (*Cryptomeria japonica*), which was collected in our university forest with permission from our university and handled in accordance with relevant guidelines and regulations. These specimens were extracted with ethanol/benzene (1:2 v/v) for 24 h in a Soxhlet apparatus. The extracted wood specimens were oven-dried at 105 °C, and their dry weights were measured.

### Preparation of IL-treated woods

To prepare IL-treated woods, the ILs were dissolved in methanol at 10 wt% concentrations. The prepared solutions were impregnated into the wood specimens at ambient temperature under reduced pressure (20 hPa) for 24 h. The impregnated specimens were then placed in an oven to dry at 60 °C for 24 h and then at 105 °C for an additional 24 h.

### Evaluations of the IL-treated woods

The weight percent gain (WPG) of each IL-treated wood specimen was determined on the basis of its oven-dried weight, as shown in Eq. (1), after measuring the oven-dried weights of extractive-free untreated specimens (Wu) and IL-treated wood specimens (Wc).

1 $${\text{WPG }}\left( \% \right) \, = \, \left( {{\text{Wc }} - {\text{ Wu}}} \right)/{\text{Wu }} \times { 1}00$$

The bulking coefficient (B) of each IL-treated wood sample was determined according to its oven-dried volume, as shown in Eq. (2), after measuring the oven-dried volumes of extractive-free untreated specimens (Vu) and IL-treated wood specimens (Vc).

2 $${\text{B }}\left( \% \right) \, = \, \left( {{\text{Vc }} - {\text{ Vu}}} \right)/{\text{Vu }} \times { 1}00$$

To test their thermal properties, samples (~ 5 mg) of the IL-treated wood specimens were studied using a simultaneous thermogravimetric and differential thermal analyzer (TG–DTA; Seiko Instruments Inc. TG/DTA 6200) with a 50 mL/min flow of dry air. The temperature was increased from room temperature to 800 °C at a heating rate of 20 °C/min.

To study the morphological changes in the samples, the IL-treated wood specimens’ surfaces were obtained with a microtome, mounted on a specimen-holder, and Pt-coated for scanning electron microscopy (SEM; JEOL JSM-5510LV) observations at an accelerating voltage of 15 kV.

The antiswelling efficiency (ASE) was determined for IL-treated wood specimens that were immersed in distilled water for 24 h. Additionally, the leachability of ILs from the corresponding IL-treated wood specimen was determined on the basis of its oven-dried weight, as shown in Eq. (3), after measuring the WPG of the IL-treated wood before soaking and the WPG of the IL-treated wood after soaking (WPG’).

3 $${\text{Leachability }}\left( \% \right) \, = \, \left( {{\text{WPG }}{-}{\text{ WPG}}^{\prime } } \right)/{\text{WPG }} \times \, 100$$

*Coptotermes formosanus* (Shiraki) and *Reticulitermes speratus* (Kolbe) were used to study the termite resistance of the IL-treated woods. The test cup used for these experiments was an acrylic cylinder (height = 50 mm, diameter = 90 mm) that was hardened with dental plaster at a thickness of approximately 5 mm. A 1-mm plastic mesh net was laid in the center of each acrylic cup, and the IL-treated wood and untreated wood specimens were placed inside one-by-one. Then, 150 worker termites and 15 soldier termites were placed in each cup. These cups were placed in a container lined with a cotton spread that was moistened with distilled water. The container was covered with a lid containing holes for ventilation and then placed in an incubator controlled at 28 ± 2 °C. The number of surviving termites was counted every few days; dead termites and mold were removed throughout the test, as needed. In addition, an appropriate amount of distilled water was supplied to the cotton every day to humidify the interior of the container.

The mortality of workers was calculated according to the number of surviving termites (N) using Eq. (4).

4 $${\text{Mortality }}\left( \% \right) \, = \, ({15}0{-}{\text{N}})/{15}0 \, \times { 1}00$$

After the termite resistance tests, the remaining treated and untreated wood specimens were oven-dried at 105 °C for 24 h and then weighed. The weight loss was calculated based on the oven-dried weight before (W1) and after (W2) the termite resistance tests using Eq. (5).

5 $${\text{Weight loss }}\left( \% \right) \, = \, ({\text{W1}}{-}{\text{W2}})/{\text{W1 }} \times { 1}00$$

## Conclusions

Wood samples were treated with various PF_6_-based ionic liquids to promote fire- and termite resistance. The prepared IL-treated wood specimens were similar in appearance to untreated wood, and no defects (e.g., distortions or cracks) were observed. SEM observations revealed that the location of the ionic liquids in the wood differed depending on the impregnated IL. Compared with untreated wood, all IL-treated woods exhibited enhanced fire resistance and termite resistance. Thus, PF_6_-based ILs were determined to be effective chemicals for enhancing fire and termite resistance of wood.

It is known that ionic liquids are nonvolatile compounds. Because the PF_6_-based ionic liquids used in this study also have negligible vapor pressure, it is thought that fluorine does not volatilize into atmosphere during treatment such as reducing pressure and drying. No data on the toxicity on PF_6_-based ionic liquids used in this study were available yet. In case of practical use of these ionic liquids for wood, however, it will be necessary to conduct environmental assessment from the viewpoint of their toxicity. Commonly, water-soluble phosphorus and/or boron-based chemicals are used for enhancing fire resistance of wood. In case that woody materials treated with these chemicals are used as construction materials, such chemicals inside wood leach out to wood surface as wood absorbs and desorbs moisture. This leaching of chemicals can cause decrease in the fire resistance of wood. While, woods treated with the ionic liquids such as [TBP]PF_6_ and [THP]PF_6_ is expected to keep their fire resistance because these ionic liquids show low leachability from wood even after immersing in water. Especially, the [TBP]PF_6_ IL is particularly promising because it is stable in wood (i.e., it does not leach from the wood after water penetration), which means that it can be expected to serve as a chemical for enhancing fire and termite resistance of wood for a long time at least for indoor use.

## Data Availability

The datasets used and/or analyzed during the current study available from the corresponding author on reasonable request.
